# FOXC1 up‐regulates the expression of toll‐like receptors in myocardial ischaemia

**DOI:** 10.1111/jcmm.14626

**Published:** 2019-09-13

**Authors:** Shao‐Ping Zhang, Ruo‐Han Yang, Jia Shang, Ting Gao, Rui Wang, Xiao‐Dong Peng, Xiao Miao, Lei Pan, Wen‐Jun Yuan, Li Lin, Qi‐Kuan Hu

**Affiliations:** ^1^ Department of Physiology, Institute of Basic Medicine Ningxia Medical University Yinchuan China; ^2^ Innovation Research Institute of Traditional Chinese Medicine Shanghai University of Traditional Chinese Medicine Shanghai China; ^3^ Department of Pharmacology, College of Pharmacy Ningxia Medical University Yinchuan China; ^4^ Department of Pharmacy First People's Hospital Guangyuan China; ^5^ Department of Physiology, School of Basic Medical Sciences Wuhan University Wuhan China; ^6^ Department of Physiology Shanghai Jiao Tong University School of Medicine Shanghai China; ^7^ Department of Cardiology Zhongshan Hospital, Fudan University Shanghai China; ^8^ Department of Physiology Second Military Medical University Shanghai China; ^9^ Key Laboratory of Arrhythmias of the Ministry of Education of China Tongji University Shanghai China

**Keywords:** FOXC, myocardial ischaemia, toll‐like receptor

## Abstract

Myocardial ischaemia (MI) remains a major cause of death and disability worldwide. Accumulating evidence suggests a significant role for innate immunity, in which the family of toll‐like receptors (TLRs) acts as an essential player. We previously reported and reviewed the changes of *Tlr* expression in models of MI. However, the underlying mechanisms regulating *Tlr* expression in MI remain unclear. The present study first screened transcription factors (TFs) that potentially regulate *Tlr* gene transcription based on in silico analyses followed by experimental verification, using both in vivo and in vitro models. Forkhead box C1 (FOXC1) was identified as a putative TF, which was highly responsive to MI. Next, by focusing on two representative TLR subtypes, an intracellular subtype TLR3 and a cell‐surface subtype TLR4, the regulation of FOXC1 on *Tlr* expression was investigated. The overexpression or knockdown of *FoxC1* was observed to up‐ or down‐regulate *Tlr*3/4 mRNA and protein levels, respectively. A dual‐luciferase assay showed that FOXC1 trans‐activated *Tlr*3/4 promoter, and a ChIP assay showed direct binding of FOXC1 to *Tlr*3/4 promoter. Last, a functional study of FOXC1 was performed, which revealed the pro‐inflammatory effects of FOXC1 and its destructive effects on infarct size and heart function in a mouse model of MI. The present study for the first time identified FOXC1 as a novel regulator of *Tlr* expression and described its function in MI.

## 1 INTRODUCTION

Ischaemic heart disease is the leading cause of death and disability in most countries worldwide. Accumulating evidence shows that innate immunity plays an essential role in myocardial ischaemia.^1^ The innate immunity, which manifests as inflammation, is activated when pattern recognition receptors (PRRs) respond to invading microbial pathogens and endogenous danger molecules, known as pathogen‐associated molecular patterns (PAMPs) and damage‐associated molecular patterns (DAMPs), respectively.^2^ The first and best characterized class of PRRs is the toll‐like receptor (TLR) family, which plays a central role in innate immune responses.^3^ In humans, 10 functional TLRs (TLR1‐TLR10) have been identified so far. The expression of TLR members has been detected in cardiomyocytes, providing novel insights into the inflammatory response initiated by cardiomyocytes themselves.^1^ A large number of studies show that TLRs in cardiomyocytes mediate cardiac inflammation and other responses to PAMPs and DAMPs. The involvement of cardiac TLRs in myocardial ischaemia has been well documented.^4^, ^5^

Changes of *Tlr* expression have been observed for myocardial ischaemia, as we reviewed previously.^1^ Fallach et al^6^ reported increased immunohistochemical staining for TLR4 in ischaemic mouse heart. Our published data showed increases in mRNAs and proteins for TLR2, TLR3 and TLR4 in cultured cardiomyocytes exposed to ischaemia, as well as heart tissue subjected to ischaemia.^7^, ^8^, ^9^ As a fact, we have examined more members of TLR family and have obtained data, which are presented herein, showing universal increases in *Tlr* mRNAs in ischaemic cardiomyocytes and myocardium. To uncover the underlying mechanism stimulating *Tlr* expression in cardiomyocytes, the present study screened transcription factors (TFs) that potentially regulate TLR gene transcription, and identified forkhead box C1 (FOXC1) as an ischaemia‐responsive TF that up‐regulates the expression of TLR members in myocardial ischaemia.

FOXC1 belongs to the FOX family of transcription factors, which is characterized by the presence of an evolutionary conserved ‘forkhead’ or ‘winged‐helix’ DNA‐binding domain.^10^ This family comprises more than 100 members in humans, classified from FOXA to FOXR on the basis of sequence similarity. FOX members participate in a wide variety of cellular processes, such as cell proliferation, differentiation, migration and metabolism.^11^ Studies on mouse mutants show that FOXC1, in cooperation with FOXC2, is required for normal embryonic development including cardiovascular development.^12^, ^13^ Consistent with the importance of *FoxC1* gene in murine development, genetic mutations and copy‐number variations of human *FoxC1* gene have been found in individuals with congenital cardiovascular defects such as mitral valve dysplasia, atrial septal defect and aortic coarctation.^12^, ^14^ The analysis of RNA isolated from human failing and non‐failing hearts suggests a role of FOXC1 in heart failure pathogenesis.^15^ Recently, FOXC1 was identified as a hypoxia‐inducible TF that plays a critical role in tumour microenvironment‐promoted lung cancer progression.^16^ However, the role of FOXC1 in myocardial ischaemia remains unclear. The present study detected significant increases of FOXC1 in in vivo and in vitro models of myocardial ischaemia and uncovered its regulation on TLR expression.

## 2 MATERIALS AND METHODS

### 2.1 Construction of FOXC1 adenoviruses and luciferase reporter plasmids

The recombinant adenovirus expressing FOXC1 was constructed from a commercial plasmid pHBAd‐EF1‐MCS‐GFP (Hanbio Biotechnology Co., Ltd). The consensus coding sequence of human *FoxC1* (gene ID: 2296) was chemically synthesized and inserted between the EcoRI and NotI sites of the pHBAd‐EF1‐MCS‐GFP vector, in which the EF1 promoter drove *FoxC1* expression and the CMV promoter drove GFP expression. The pHBAd‐EF1‐MCS‐GFP vector harbouring *FoxC1* was then cotransfected with the backbone vector pHBAd‐BHG into HEK293 cells. The recombinant adenovirus was harvested and purified using a standard protocol,^17^ and the infectious titre in plaque‐forming units (pfu)/mL was calculated from the 50% cell culture infective dose (CCID 50) assay.^18^

To assay transcriptional activity of *Tlr* genes, the pGL3‐Basic plasmids that contain a modified coding region for firefly luciferase were used to construct reporter vectors. The proximal promoter sequences (−2000‐+1 bp) of human *Tlr*3 (ID: 7098) and *Tlr*4 (ID: 7099) genes were chemically synthesized and separately cloned into the KpnI and NheI sites of the pGL3‐Basic plasmids.

### 2.2 Mice model of myocardial infarction

Mice (8 ~ 10 weeks of age) were purchased from SIPPR‐BK Laboratory Animal Co. Ltd., Shanghai, China, and the model of myocardial infarction was prepared as we described previously.^7^ Briefly, mice were initially anaesthetized in an induction chamber filled with isoflurane at 3%‐4% and then maintained via a nose cone with 2% isoflurane in oxygen at 1.5 L/min. The adequacy of anaesthesia was checked by the lack of corneal reflex and withdrawal reflex to toe pinch. The chest was depilated, a small skin cut was made on the left, and a small hole was made under the fourth rib using a mosquito clamp. The clamp was slightly opened to ‘pop out’ the heart through the hole. Then, the left anterior descending coronary artery (LAD) was sutured and ligated with a 6/0 braided silk suture. Infarction was confirmed by visual cyanosis. After ligation, the heart was immediately placed back into the intrathoracic space, and the chest was closed. Then, the mouse was started to breathe room air and monitored until recovery. Sham mice received the same procedure except that LAD was not ligated.

To overexpress *FoxC1* in heart tissue, 30 μL of normal saline (NS) containing 5 × 10^9^ pfu/mL adenoviruses was directly injected into the left ventricle at 3 spots around the infarct border, just after LAD ligation, using a 33G needle (Hamildon).^8^, ^19^ To suppress *FoxC1* expression, the small interference RNA (siRNA) against *FoxC1* was delivered in a similar way into the myocardium at the dose of 4.5 nmol/heart, using in vivo‐jetPEI delivery reagent (Genesee Scientific). Otherwise, vehicle solution was injected as control. After that, the heart was gently restored to their normal anatomic position; then, the chest was closed.

At the end of the 2‐week observation period and after echocardiography, the mice were killed by placing into a chamber filled with vapour of isoflurane, and heart tissue was then collected for examination.

All animal procedures were approved by the Animal Experiment Committee of Ningxia Medical University, in accordance with the Guide for the Care and Use of Laboratory Animals published by the US National Institutes of Health (8th Edition, 2011).

### 2.3 Cell culture and treatments

The neonatal rat ventricular myocytes (NRVMs) and H9c2 rat ventricular cell line were cultured as we described previously.^7^, ^20^ NRVMs were prepared from neonatal Sprague Dawley rats. Briefly, the neonatal rats were killed by decapitation; then, the ventricles were removed, rinsed, minced and digested with 0.2% trypsin in Ca^2+^‐ and Mg^2+^‐free Hanks solution for repeated short time periods. After digestion, cells were collected and resuspended in Dulbecco's Modified Eagle Medium (DMEM) supplemented with foetal bovine serum, pre‐plated and then cultured in a humidified atmosphere of 95% air and 5% CO_2_ at 37°C. To mimic ischaemia, cells were exposed to 1% O_2_ ‐ 94% N_2_ ‐ 5% CO_2_ in serum‐free low‐glucose DMEM for 24 hours.

To overexpress *FoxC1*, adenoviruses were incubated overnight at 3 × 10^7^ pfu/mL prior to the onset of ischaemia. To knockdown *FoxC1*, cells were transfected with 12.5 nmol/L of siRNA duplexes against *FoxC1*, with the aid of Lipofectamine^®^ RNAiMAX reagent. The sequences of siRNAs are as following: negative control (NC): sense 5′‐UUCUCCGAACGUGUCACGUTT‐3′, antisense 5′‐ACGUGACACGUUCGGAGAATT‐3′; *FoxC1* siRNA: sense 5′‐CCACGUAAGUUUCUUGCGUTT‐3′, antisense 5′‐ACGCAAGAAACUUACGUGGTT‐3′. All the reagents for cell isolation, culture and transfection were purchased from Thermo Fisher Scientific Inc.

### 2.4 In silico analyses of transcription factors

The gene information for human/mouse/rat *Tlr*1‐9 was individually entered on the website http://genome.ucsc.edu/cgi-bin/hgNear to get the promoter sequence of each TLR gene. Candidate TFs that potentially bind to all the promoter sequences of *Tlr*1‐9 genes of the same species were predicted using the JASPAR database (http://jaspar.binf.ku.dk), and Venn diagram was plotted to identify the shared TFs among different species. Then, the tissue distribution characteristics of the shared TFs were checked through two databases (http://www.uniprot.org and http://www.proteinatlas.org). Those abundantly expressed in the heart were further screened by changes upon ischaemia.

### 2.5 Real‐time reverse transcription‐polymerase chain reaction (RT‐PCR)

The mRNA levels of target genes were measured by real‐time RT‐PCR analysis. Total RNA was extracted with TRIzol reagent (Invitrogen), following the manufacturer's instructions. Approximately 4 μg of total RNA from each sample was reverse transcribed into cDNA using a cDNA synthesis kit (Thermo Fisher Scientific). The acquired cDNA was used as template to run real‐time PCR reactions, using the SYBR^®^ Premix Ex Taq II Kit (Takara). All reactions were performed in duplicate. The primer sets for real‐time PCR are listed in Table S1. The relative level of target mRNA was calculated by the method of 2^−∆∆^ *^C^* ^t^, with 18S ribosomal RNA serving as the loading control.

### 2.6 Western blot assay

Heart tissue and cultured cardiomyocytes were lysed in RIPA buffer containing protease inhibitors at 4°C. Lysates were collected and determined with protein concentration using a bicinchoninic acid kit. For Western blot, 20 µg of total proteins was separated on the SDS‐PAGE gel and transferred onto PVDF membrane (Immobilon‐P Transfer Membrane, Millipore Corp). The membrane was then blocked with PBST buffer containing 5% non‐fat dried milk for 1 hour and further incubated overnight at 4°C with primary antibodies against TLR3 (ab62566, Abcam), TLR4 (NB100‐56566, NOVUS) or FOXC1 (#8758, Cell Signaling Technology). After that, the membrane was incubated with horseradish peroxidase (HRP)‐conjugated secondary antibodies (sc‐2004, sc‐2005, Santa Cruz Biotechnology) for 1.5 hours at room temperature and visualized by chemiluminescence reagents. GAPDH (ab8245, Abcam) was used as a loading control.

### 2.7 Echocardiographic examination

Transthoracic echocardiography was performed under the anaesthesia of isoflurane, using a VisualSonics 2100 system equipped with a MS400 linear array transducer (30 MHz). The short‐axis view was acquired at the papillary muscle level of the heart through two‐dimensional mode, and consecutive M‐mode images were then recorded. Left ventricular end‐diastolic diameter (LVEDD) and end‐systolic diameter (LVESD) were measured from M‐mode tracings, and fractional shortening (FS) was calculated as (LVEDD‐LVESD)/LVEDD*100%.

### 2.8 Masson's trichrome staining and infarct size measurement

After euthanasia, the mouse heart was isolated, retrogradely perfused with cold normal saline followed by 4% paraformaldehyde for fixation, dehydrated with ethanol, embedded in paraffin, coronally sectioned into 5‐μm‐thick slices and then stained with Masson's trichrome reagents.^7^ After staining, collagen fibres were blue, while muscle fibres were purple‐red.

Using Masson's images, infarct size was calculated as the percentage of midline infarct length relative to LV circumference, according to a length‐based approach.^7^, ^21^ LV circumference was taken as the length of LV midline, which was drawn at the centre between the epicardial and endocardial surfaces of LV. Midline infarct length was taken as the midline of the length of infarct that included >50% of wall thickness.

The method of triphenyl‐tetrazolium chloride (TTC) staining was also used to measure infarct size. Briefly, 1% Evan's blue dye was retrogradely infused into the heart through the aorta to identify the risk area (non‐blue); then, the heart was transversely cut into ~2 mm thick slices, followed by incubation in 1% TTC (Sigma‐Aldrich Corp) at 37°C for 15 minutes. The viable tissue was stained red by TTC, while the infarct tissue was not stained. The infarct area (white) and the risk area (non‐blue) were then calculated by ImageJ, and the infarct size was calculated as a percentage of the infarct area vs the risk area.

### 2.9 Luciferase reporter assay

H9c2 cells seeded in 24‐well plates were used for dual‐luciferase assay. The cells were infected with *FoxC1* or control adenovirus for 12 hours. Then, the pGL3 reporter vector that contained *Tlr*3/4 promoter driving firefly luciferase was cotransfected with phRL‐SV40 plasmid expressing Renilla luciferase, at the final concentration of 40 nmol/L and 4 nmol/L, respectively. The cotransfection was performed with Lipofectamine™ 2000, according to the manufacturer's instructions (Thermo Scientific). After 24 hours, cells were eventually lysed for analysis with dual‐luciferase assay reagents (Promega). Firefly luciferase activities were measured on a GloMax^®^ 20/20 luminometer (Promega) and normalized to those of Renilla luciferase.

### 2.10 Chromatin immunoprecipitation assay

Chromatin immunoprecipitation (ChIP) assay was performed on mouse heart tissue to examine the binding of FOXC1 to *Tlr* promoter sequences, using the EZ‐ChIP kit (#9005, Cell Signaling Technology, Inc). Approximately 25 mg of normal or ischaemic tissue was ground in liquid nitrogen, added with PBS containing protease inhibitor cocktail, subjected to protein‐DNA cross‐link in 1% formaldehyde at 37°C for 10 minutes and then quenched with 0.125 M Glycine for 5 minutes at room temperature. After washing with PBS, the homogenates were sonicated to shear DNA into fragments of 100 ~ 500 bp. The protein‐DNA complexes were then immunoprecipitated with anti‐FOXC1 antibodies (ab5079, Abcam Inc) in the presence of herring sperm DNA and protein G beads, and isotype IgG was used as the negative control. The immunoprecipitated DNA was then retrieved from the beads, purified and analysed with real‐time PCR. The putative binding sites with top 3 scores (Table S2 and S3) were probed with specific primers (Table S4). Sonicated DNA from the same sample that had not been precipitated with the antibody, commonly called ‘Input’, was also performed with PCR for data normalization. % Input was calculated to represent the % of DNA being precipitated by the target antibody.

### 2.11 Analyses of human expression data

The expression data of TLRs and the candidate TFs in patients with ischaemic cardiomyopathy were looked up in published data sets on Gene Expression Omnibus (GEO). Three data sets (GSE1145, GSE1869 and GSE5406) that contain normal and ischaemic heart gene expression values were downloaded. The differences in expression values were compared between normal and ischaemic groups by unpaired *t* test, and the results were shown in Figure S1.

### 2.12 Data analyses and statistics

All data are expressed as mean ± SEM. The parameters of multiple groups were analysed by the one‐way analysis of variance (ANOVA) followed by the Dunnett's test, and the parameters of two groups were analysed by unpaired *t* test, using SAS 9.0 statistical software (SAS Institute Inc). Two‐tailed *P* values <.05 were considered statistically significant.

## 3 RESULTS

### 3.1 Up‐regulation of Tlr mRNAs in models of myocardial ischaemia

**Figure 1:**
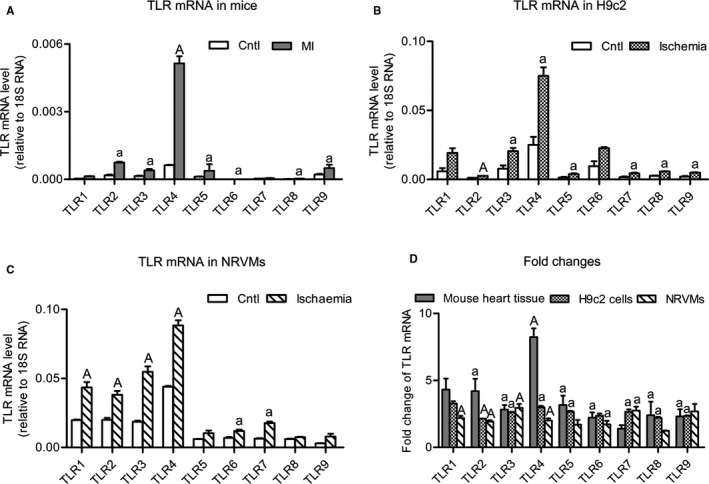
Increases in toll‐like receptor (TLR) mRNA levels in myocardial ischaemia. The mRNA levels of TLR1‐9 in ischaemia models for mice (A), H9c2 cells (B) and neonatal rat ventricular myocytes (NRVMs) (C) were determined, and fold changes were calculated (D). The mouse model of myocardial ischaemia (MI) was caused by left coronary ligation, and the left ventricle was sampled for examination after 2 wk. Ischaemia of H9c2 and NRVMs cells was induced by 1% O_2_ combined with serum‐free low‐glucose DMEM for 24 h. The mRNA levels were determined by real‐time RT‐PCR and normalized to 18S ribosome RNA. Data are means ± SEM. n = ~4‐5/group. ^a^ *P* < .05, ^A^ *P* < .01 vs control

To reveal changes in *Tlr* mRNA expression caused by myocardial ischaemia, we examined mRNAs for *Tlr*1‐9 in a murine myocardial infarction model, and in cultured H9c2 myocytes and NRVMs exposed to ischaemia. The results (Figure 1) showed that the mRNA levels of multiple *Tlr*s were up‐regulated in these models of myocardial ischaemia. *Tlr*4 mRNA exhibited the largest increase in ischaemic myocardium. Based on the consideration of mRNA abundance, fold change and subcellular localization, an intracellular subtype TLR3 and a cell‐surface subtype TLR4 were selected as representative TLR members for downstream experiments.

### 3.2 Screening and analyses of transcription factors potentially regulating TLR expression

**Figure 2:**
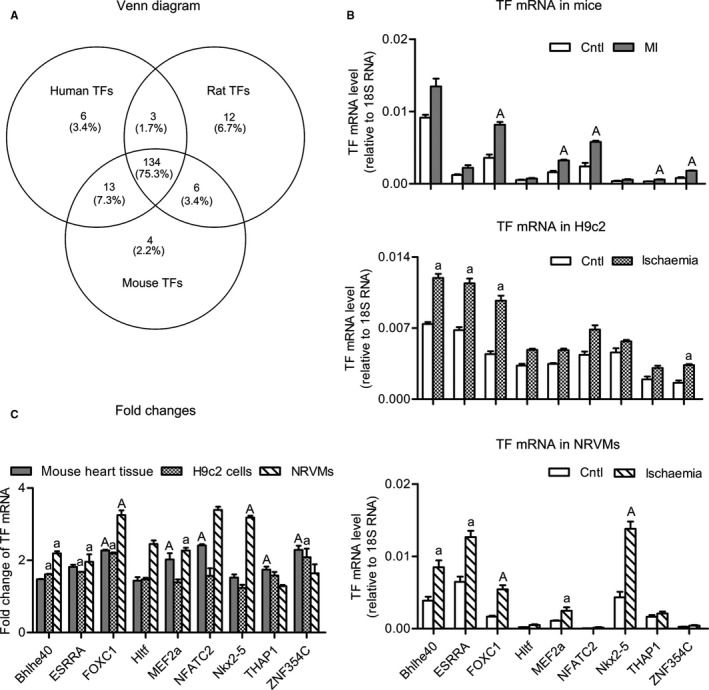
Screening and analysis of transcription factors (TFs) that potentially regulate TLR expression. A, Venn diagram showing the number of TFs in humans, mice and rats predicted to bind with *Tlr*1‐9 promoters. B, The mRNA levels of selected TFs in ischaemia models for mice (upper panel), H9c2 cells (middle panel) and NRVMs (lower panel). C, Fold changes of mRNA levels in each model. Data are means ± SEM. n = ~4‐5/group. ^a^ *P* < .05, ^A^ *P* < .01 vs control

We hypothesized that the general increases in *Tlr* mRNAs were controlled by an ischaemia‐responsive TF. To identify that TF, we employed an in silico approach for the first step. We input the promoter sequences of *Tlr*1‐9 genes into the JASPAR database (http://jaspar.binf.ku.dk) and searched for TFs that potentially bind with all the promoters. It turned out that 156 TFs in humans, 157 TFs in mice and 155 TFs in rats were found. Using an online tool of plotting Venn diagram (http://bioinfogp.cnb.csic.es/tools/venny/index.html), 134 TFs were identified to be common among different species (Figure 2A). We further checked the tissue distribution of these TFs on UniProt database (http://www.uniprot.org) and human protein atlas (http://www.proteinatlas.org), and 9 TFs that abundantly expressed in the heart were selected for further determination of expression changes upon ischaemia.

Using the method of real‐time RT‐PCR, the mRNA levels of the 9 selected TFs were determined for the aforementioned three models of myocardial ischaemia (Figure 2B and 2C). Overall, *FoxC1* mRNA exhibited high basal expression and large increases under ischaemia. Compared with non‐ischaemic myocardium from sham‐operated mouse heart, *FoxC1* mRNA in the ischaemic myocardium was increased by 2.3 ± 0.3‐fold. In H9c2 myocytes and NRVMs, *FoxC1* mRNA was increased by 2.2 ± 0.2‐fold and 3.3 ± 0.6‐fold, respectively. Based on these data, FOXC1 was selected for further examining its regulation on *Tlr* genes.

From the publicly available data sets on GEO, we found three data sets that contain human expression data of *Tlr*s and the selected TFs, under the GEO accession numbers GSE1145 (Martina et al), GSE1869 (Kittleson et al) and GSE5406 (Cappola et al). These data sets were from microarray assays, showing gene expression profiles in the left ventricular tissue of patients with ischaemic cardiomyopathy. The changes in *Tlr*s and the selected TFs were not consistent between different studies. The GSE1145 data set showed up‐regulation of *Tlr3/7* and *FoxC1*, and the GSE5406 data set showed up‐regulation of *Tlr4* (Figure S1). The discrepancies between data sets may be derived partially from differences in study populations and from the variability characterization of microarray, and the discrepancies with our study may at least in part be due to differences in species and in testing methods.

### 3.3 Regulation of FOXC1 on TLR expression

**Figure 3:**
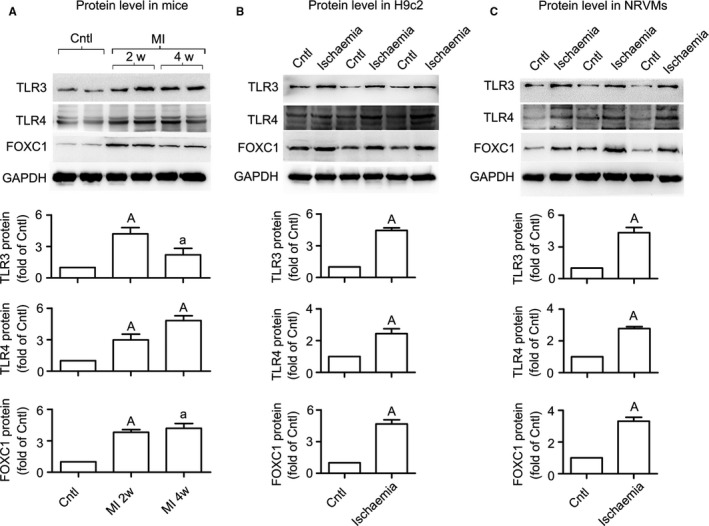
Up‐regulation of FOXC1 and TLR3/4 protein levels under myocardial ischaemia. Representative Western blot images and quantitative data for FOXC1, TLR3 and TLR4 proteins in ischaemia models of mice (A), H9c2 cells (B) and NRVMs (C) are shown herein. Validation data of the antibodies for TLR3 and TLR4 are shown in Figure S1. Data are means ± SEM. n = ~4‐5/group. *P* values from the one‐way ANOVAs: 0.018 (TLR3 protein in mice), .002 (TLR4 protein in mice) and 0.006 (FOXC1 protein in mice). ^a^ *P* < .05, ^A^ *P* < .01 vs control

To clarify the expression profile of *FoxC1* and *Tlr*3/4 in response to myocardial ischaemia, we further examined protein levels for FOXC1 and TLR3/4. The results showed that, in line with increased mRNAs for *FoxC1* and *Tlr*3/4, their proteins were all increased upon ischaemia (Figure 3). These data suggest that the expression of both *FoxC1* and TLR subtypes is up‐regulated under ischaemia.

**Figure 4:**
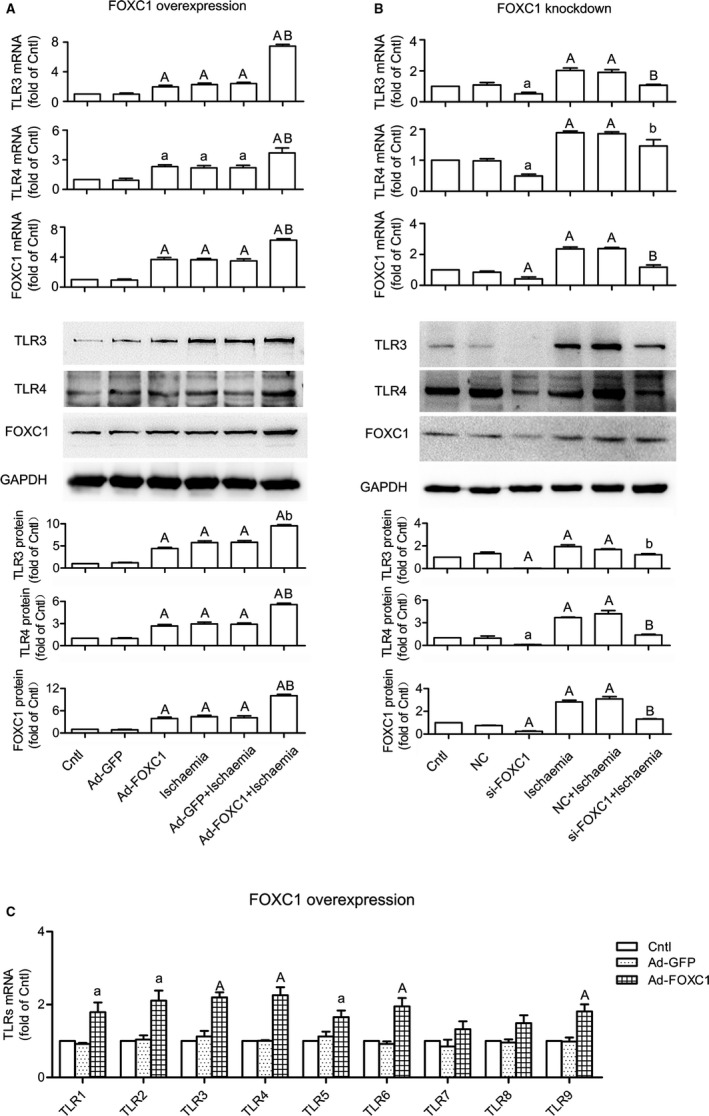
Regulation of FOXC1 on TLR expression. H9c2 cells were transfected with adenovirus or siRNA to overexpress or knock down *FoxC1*, with GFP adenovirus (Ad‐GFP) and negative control (NC) siRNA serving as control, respectively. A, *FoxC1* overexpression increased *Tlr*3/4 mRNA and protein levels, under both control and ischaemic conditions. *P* values from the one‐way ANOVAs: <.001 (TLR3 mRNA), .002 (TLR4 mRNA), <.001 (FOXC1 mRNA), <.001 (TLR3 protein), <0.001 (TLR4 protein) and <.001 (FOXC1 protein). B, *FoxC1* knockdown decreased *Tlr*3/4 mRNA and protein levels. *P* values from the one‐way ANOVAs: .001 (TLR3 mRNA), <.001 (TLR4 mRNA), <.001 (FOXC1 mRNA), .002 (TLR3 protein), <.001 (TLR4 protein) and <.001 (FOXC1 protein). C, *FoxC1* overexpression up‐regulated the mRNA expression of multiple *Tlr* subtypes. *P* values from the one‐way ANOVAs: .022 (TLR1 mRNA), .047 (TLR2 mRNA), .002 (TLR3 mRNA), .013 (TLR4 mRNA), .039 (TLR5 mRNA), .003 (TLR6 mRNA), .203 (TLR7 mRNA), .078 (TLR8 mRNA) and .009 (TLR9 mRNA). Data are means ± SEM of 4 independent experiments. ^a^ *P* < .05, ^A^ *P* < .01 vs. control; ^b^ *P* < .05, ^B^ *P* < 0.01 vs. ischaemia

To examine whether FOXC1 regulates *Tlr* expression, *FoxC1* was either overexpressed (Figure 4A) or knocked down (Figure 4B) in H9c2 myocytes, and *Tlr*3/4 expression was examined. The results showed that, in both control and ischaemic cells, adenovirus overexpressing *FoxC1* significantly increased *Tlr*3/4 mRNA and protein levels, whereas siRNA against *FoxC1* suppressed *Tlr*3/4 expression. In addition to *Tlr*3/4, a further examination showed that FOXC1 adenovirus resulted in general increases in mRNAs for multiple *Tlr* subtypes, including *Tlr*1/2/5/6/9 (Figure 4C). These data suggest that FOXC1 regulates *Tlr* expression in myocardial ischaemia.

### 3.4 FOXC1 binds and activates TLR3/4 promoter

**Figure 5:**
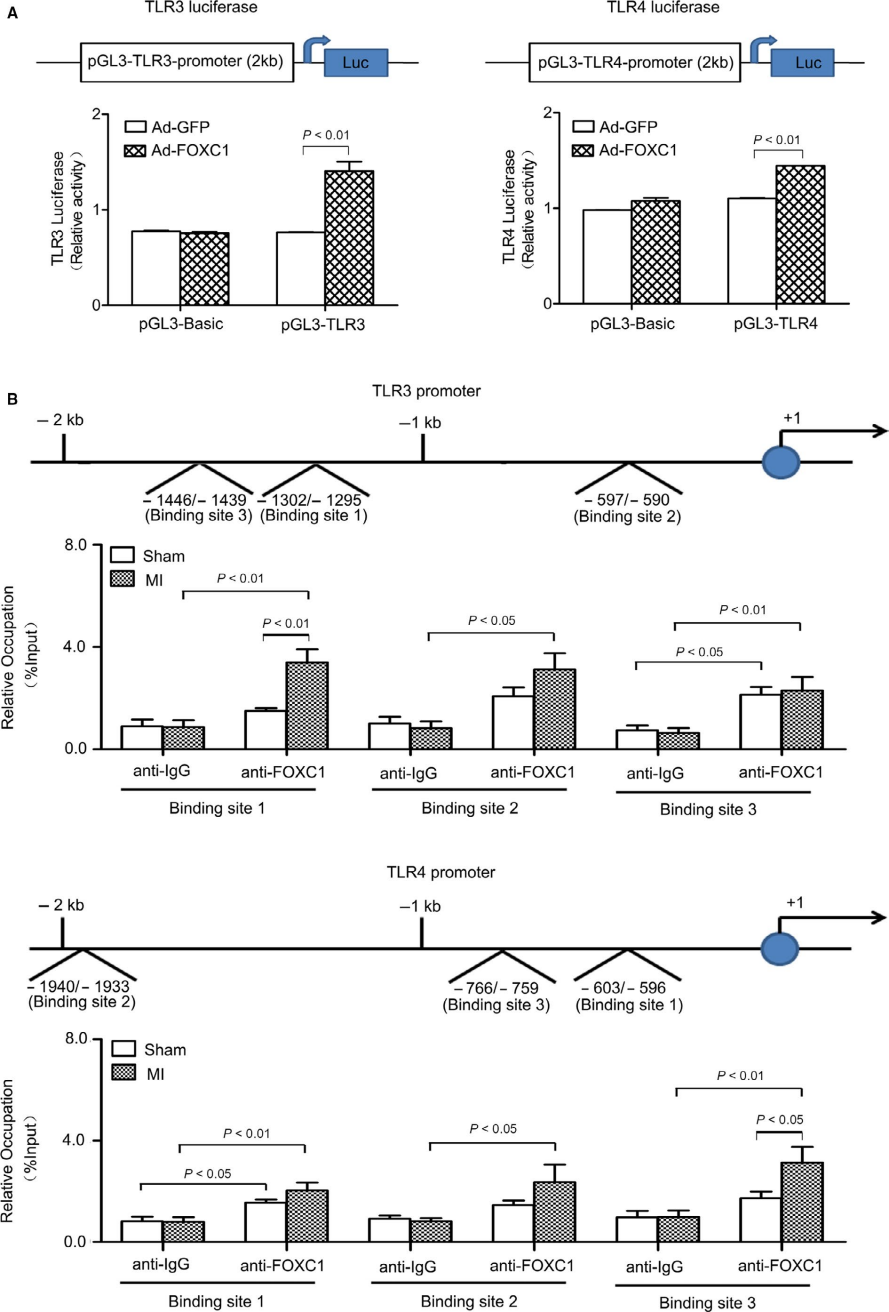
Direct binding and regulation of *Tlr*3/4 promoter by FOXC1. A, Dual‐luciferase assay performed on H9c2 cells. The pGL3 firefly luciferase reporter vector harbouring the promoter sequence (−2000‐+1 bp) of human *Tlr*3 or *Tlr*4 was constructed. The cells were cotransfected with recombinant pGL3 vector and phRL‐SV40 plasmid expressing Renilla luciferase. Firefly luciferase activities were expressed as folds of Renilla luciferase activities. The effects of FOXC1 adenovirus (Ad‐FOXC1) on firefly luciferase activities were examined. *P* values from the one‐way ANOVAs: <.001 (TLR3 luciferase) and <.001 (TLR4 luciferase). ^A^ *P* < .01 vs Ad‐GFP. B, ChIP assay performed on mouse heart tissue. Briefly, DNA was cross‐linked with proteins, fragmented, immunoprecipitated with anti‐FOXC1 or anti‐IgG antibodies, retrieved and then subjected to PCR with primers probing the top 3 putative binding sites, whose sequence and location are shown in Table S2 and S3. Data were collected from 4 independent experiments and expressed as means ± SEM *P* values from the one‐way ANOVAs: 0.001 (binding site 1 in TLR3 promoter), .015 (binding site 2 in TLR3 promoter), .011 (binding site 3 in TLR3 promoter), .007 (binding site 1 in TLR4 promoter), .067 (binding site 2 in TLR4 promoter) and .009 (binding site 3 in TLR4 promoter)

To examine whether FOXC1 trans‐activates *Tlr* promoter activity, a dual‐luciferase assay was performed on H9c2 cells. As shown in Figure 5A, in cells cotransfected with pGL3 vector harbouring *Tlr*3/4 promoter sequence and adenovirus expressing FOXC1, the firefly luciferase activity was significantly enhanced. In contrast, no changes in luciferase activity were observed for the control vector (pGL3‐Basic) or the control adenovirus (Ad‐GFP). These data suggest that FOXC1 trans‐activates *Tlr*3/4 promoter and increases their expression at the transcriptional level.

To further examine the potential binding between FOXC1 and *Tlr* gene promoter, a ChIP assay was performed on mouse heart tissue. In the DNA fragments pulled down by anti‐FOXC1 antibodies, promoter sequences of *Tlr*3 and *Tlr*4 were detected (Figure 5B). The base sequence and location of putative FOXC1 binding sites in mouse *Tlr*3 and *Tlr*4 promoters are shown in Table S2 and S3. Individual real‐time PCR reactions were performed with primers probing the top 3 putative binding sites (namely site 1, 2 and 3). Among the tested binding sites, site 3 in *Tlr*3 promoter and site 1 in *Tlr*4 promoter bound with FOXC1 under normal conditions. Sites 1 and 2 in *Tlr*3 promoter and sites 1, 2 and 3 in *Tlr*4 promoter showed more abundant binding with FOXC1 under ischaemia. These data suggest that *Tlr*3 and *Tlr*4 are direct transcriptional targets of FOXC1.

### 3.5 Pro‐inflammatory and detrimental effects of FOXC1 activation in myocardial ischaemia

**Figure 6:**
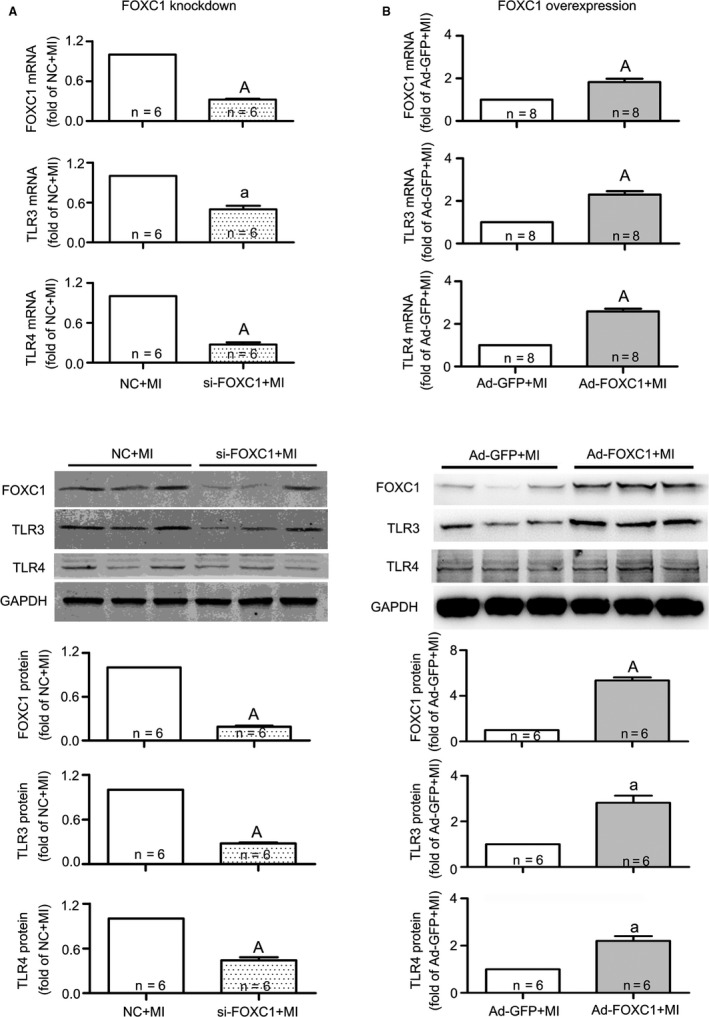
Regulation of FOXC1 on TLR3/4 expression in mice subjected to myocardial ischaemia (MI). The siRNA against FOXC1 (si‐FOXC1) or FOXC1 adenovirus (Ad‐FOXC1) was injected into the left ventricle just after LAD ligation to generate FOXC1 knockdown (A) or FOXC1 overexpression (B), and the negative control (NC) siRNA and Ad‐GFP served as control, respectively. The mRNA (upper panel) and protein (middle and lower panel) levels of TLR3/4 were determined after 2 wk. Data are means ± SEM ^a^ *P* < .05, ^A^ *P* < .01 vs respective MI

**Figure 7:**
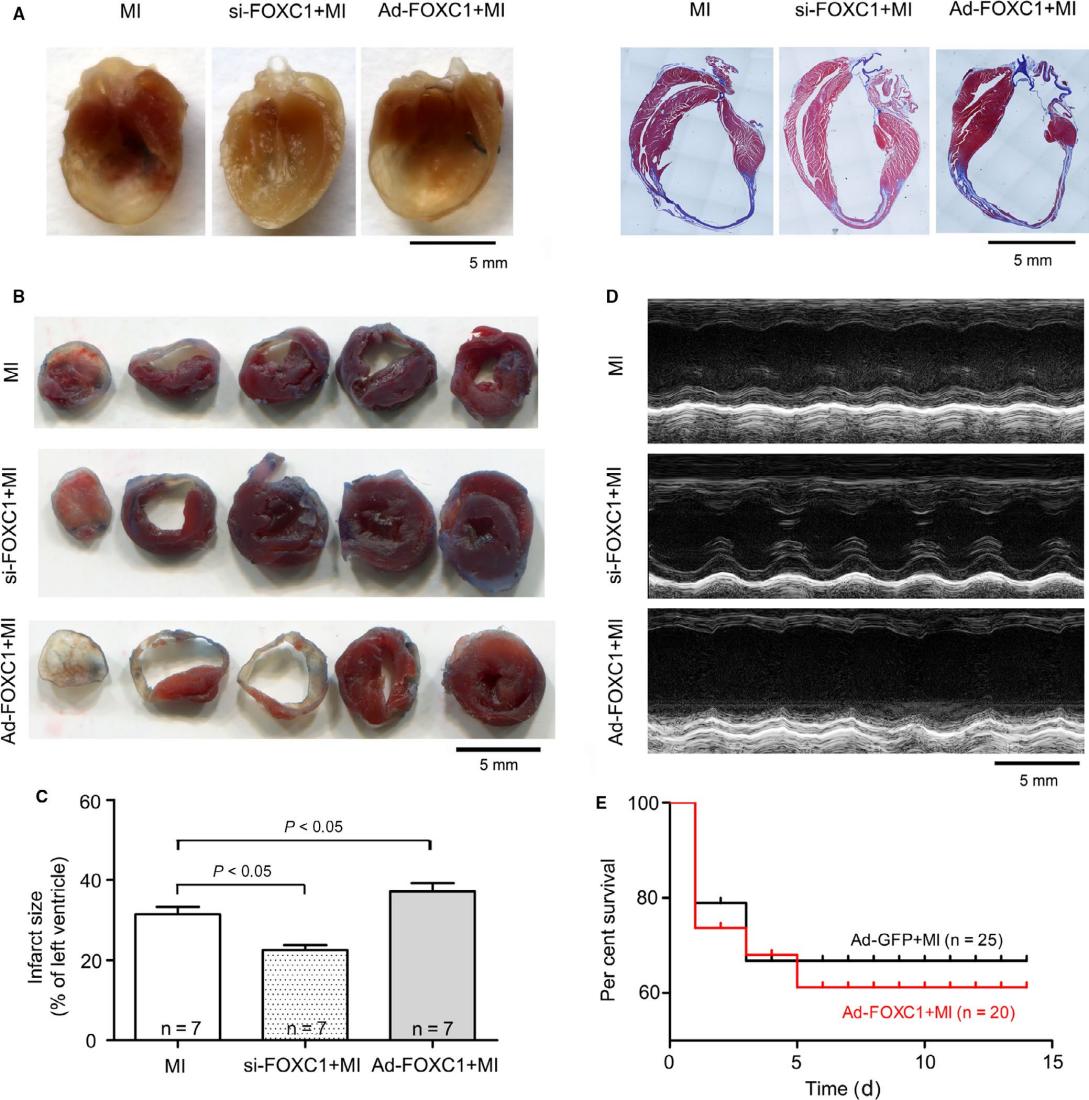
Protective effects of FOXC1 knockdown and destructive effects of FOXC1 overexpression in mice subjected to myocardial ischaemia (MI). The siRNA against FOXC1 (si‐FOXC1) or FOXC1 adenovirus (Ad‐FOXC1) was injected into the left ventricle just after LAD ligation, and the negative control (NC) siRNA and Ad‐GFP served as control, respectively. A, Representative gross view (left panel) and Masson's trichrome images (right panel) of coronally sectioned mouse heart. B, Representative images of TTC staining. C, Infarct size, expressed as means ± SEM. The *P* value from the one‐way ANOVA is .028. D, Representative M‐mode ultrasound tracings taken at the midpapillary level. (E) Kaplan‐Meier survival curves after coronary ligation surgery

By overexpressing or knocking down *FoxC1*, the functional role of FOXC1 in myocardial ischaemia was investigated. In the mouse model of myocardial ischaemia, knockdown of *FoxC1* in heart tissue substantially suppressed the mRNA and protein levels of TLR3 and TLR4 (Figure 6), reduced the infarct size and improved heart function (Figure 7 and Table S5). In contrast, myocardial overexpression of *FoxC1* increased *Tlr*3/4 mRNA and protein levels, and worsened heart function. The survival rate showed a tendency to decrease, though no significance was detected (Figure 7E). As to sham‐operated mice, neither the overexpression nor the knockdown of *FoxC1* in normal hearts caused significant changes in histology and heart function (Figure S3 and Table S5).

**Figure 8:**
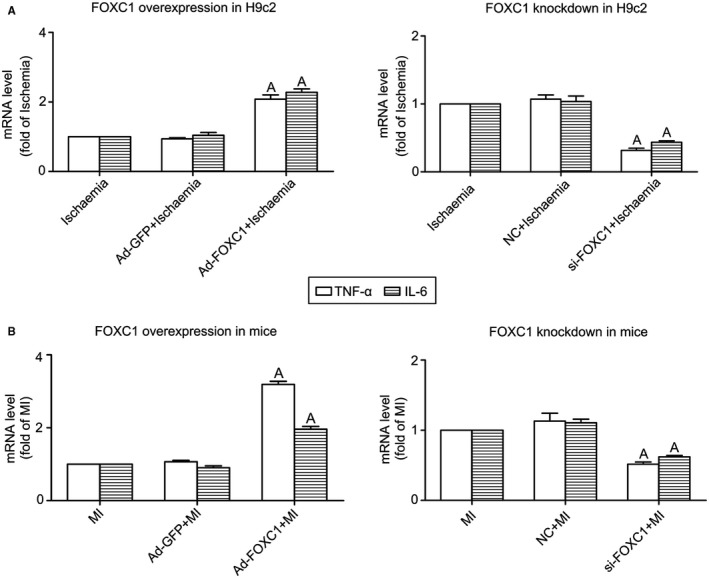
Effects of FOXC1 overexpression and knockdown on inflammatory cytokine expression. The mRNA levels of inflammatory cytokine markers tumour necrosis factor α (TNFα) and interleukin‐6 (IL‐6) were determined in (A) H9c2 cells and (B) left ventricle tissue of a mouse model of myocardial ischaemia (MI), using real‐time RT‐PCR. The effects of FOXC1 adenovirus (Ad‐FOXC1) and FOXC1 siRNA (si‐FOXC1) were measured, with Ad‐GFP and NC siRNA serving as control, respectively. Data are means ± SEM. n = ~4‐5/group. ^A^ *P* < 0.01 vs respective Ischaemia/MI *P* values from the one‐way ANOVAs: 0.013 (TNFα, FOXC1 overexpression in H9c2), <.001 (IL‐6, FOXC1 overexpression in H9c2), .003 (TNFα, FOXC1 knockdown in H9c2), <.001 (IL‐6, FOXC1 knockdown in H9c2). <.001 (TNFα, FOXC1 overexpression in mice), <.001 (IL‐6, FOXC1 overexpression in mice), .002 (TNFα, FOXC1 knockdown in mice) and .001 (IL‐6, FOXC1 knockdown in mice)

Considering that the activation of TLRs essentially contributes to inflammation, we further examined whether *FoxC1* has effects on inflammation. The results (Figure 8) showed that, in both H9c2 cells and the mouse model of myocardial ischaemia, *FoxC1* adenovirus significantly enhanced the expression of inflammatory cytokine markers tumour necrosis factor α (TNFα) and interleukin‐6 (IL‐6), whereas *FoxC1* siRNA reduced their expression.

## 4 DISCUSSION

The present study tried to look for TFs that regulate *Tlr* expression in myocardial ischaemia. Firstly, we screened TFs that have putative binding sites in *Tlr* promoter sequences and examined the expression of selected TFs under myocardial ischaemia. FOXC1 was found to act as an ischaemia‐responsive TF that potentially regulates *Tlr* expression. Secondly, by manipulating *FoxC1* expression in cultured cardiomyocytes, we observed that overexpression of *FoxC1* stimulated *Tlr* expression, whereas knockdown of *FoxC1* inhibited *Tlr* expression. Thirdly, by performing ChIP and luciferase assay, we verified that FOXC1 binds to *Tlr* promoter sequences and regulates *Tlr* gene expression at the transcriptional level. Lastly, by manipulating cardiac expression of *FoxC1*, we observed that the activation of FOXC1 promoted cardiac inflammation and played a detrimental role in myocardial ischaemia.

TLRs are a family of innate immune receptors that are essential for mediating innate immune responses. They are type I transmembrane proteins composed of a leucine‐rich repeat (LRR) ectodomain, a single transmembrane domain, and a cytoplasmic domain known as toll/IL‐1 receptor (TIR) domain. Upon activation, TLRs recruit TIR‐domain–containing adaptor proteins such as myeloid differentiation primary‐response gene 88 (MyD88) and TIR‐domain–containing adaptor protein inducing interferon‐β (TRIF), leading to the activation of nuclear factor‐kappa B (NF‐κB) and the induction of inflammatory responses.^3^ TLRs are expressed by a variety of immune and non‐immune cells including cardiomyocytes. In addition to generating inflammatory effects, the activation of TLRs in cardiomyocytes has multiple effects on cellular activities, including cell contractility, apoptosis and autophagy.^1^, ^7^ The contribution of cardiac TLRs typically manifested in inflammatory heart diseases, including myocardial ischaemia/reperfusion, viral myocarditis and septic cardiomyopathy. For example, as has been reviewed,^1^, ^22^ mice deficient in *Tlr*2 or *Tlr*4 exhibited less inflammation and smaller infarct size after myocardial ischaemia.

Previous studies have observed changes of *Tlr* expression under myocardial ischaemia.^1^ However, the underlying mechanism controlling *Tlr* expression in cardiomyocytes has hardly been addressed. As TFs are essential for the control of gene transcription, the present study screened TFs that potentially regulate *Tlr* gene transcription, based on database analyses. Nine TFs (Bhlhe40, ESRRA, FOXC1, Hltf, MEF2a, NFATC2, Nkx2‐5, THAP1 and ZNF354C) were then selected for examining their expression profiles. It turned out that FOXC1 exhibited most abundant expression under basal condition and in response to myocardial ischaemia. Therefore, FOXC1 was picked out for further study.

FOXC1 is a member of the FOX family which is widely involved in cellular activities.^11^ Previous studies showed that FOXC1 plays a role in regulating heart development at the embryonic stage.^12^, ^13^ Defects in *FoxC1* gene may contribute to the pathogenesis of congenital heart defects.^14^ A transcriptional genomics study suggests that FOXC1 is involved in human heart failure due to ischaemic or idiopathic dilated cardiomyopathy.^15^ A number of studies also showed that FOXC1 plays a significant role in cancer diseases, such as lung cancer, hepatocellular carcinoma, basal‐like breast cancer and endometrial cancer.^16^, ^23^, ^24^, ^25^ A recent study performed on lung cancer cells explained why *FoxC1* is up‐regulated in tumour microenvironment.^16^ It showed that *FoxC1* expression is inducible by hypoxia, which is ascribed to the direct binding of hypoxia‐inducible factor‐1α (HIF‐1α) to the hypoxia‐responsive element (HRE) in *FoxC1* promoter.^16^ In accordance with this study, we observed that *FoxC1* was up‐regulated by myocardial ischaemia.

To reveal the expression profiles under myocardial ischaemia, we determined the mRNAs for *FoxC1* and *Tlr*1‐9, and protein levels of FOXC1 and two representative TLR subtypes, TLR3 and TLR4, in both in vivo and in vitro models. Our results showed that the expression of *FoxC1* and *Tlr*s was increased upon ischaemia. High levels of FOXC1 were accompanied by high levels of TLRs and vice versa. These data supported our hypothesis that FOXC1 potentially regulates *Tlr* expression. To further prove this hypothesis, we overexpressed and knocked down *FoxC1* in cardiomyocytes (Figure 4). As a result, *Tlr*3/4 mRNA and protein levels were up‐ and down‐regulated, respectively. Additionally, the mRNAs for *Tlr*1/2/5/6/9 were observed to be up‐regulated by FOXC1 overexpression. It is demonstrated that FOXC1 regulates *Tlr* expression in cardiomyocytes.

A defining feature of TFs is that they regulate the rate of target gene transcription by binding to the promoter sequences of the genes.^26^ In the present study, a dual‐luciferase assay revealed that FOXC1 trans‐activated *Tlr*3/4 promoter activity, suggesting that FOXC1 enhances *Tlr*3/4 expression at the transcriptional level. Furthermore, a ChIP assay was performed to examine the direct binding of FOXC1 to *Tlr*3/4 promoters. The top 3 of predicted binding sites were examined, and constitutive binding was detected between site 3 in *Tlr*3 promoter and site 1 in *Tlr*4 promoter. Myocardial ischaemia significantly increased the binding of FOXC1 to *Tlr*3/4 promoter at nearly all the tested sites. These data demonstrate that FOXC1 directly binds and activates *Tlr*3/4 gene transcription under the condition of myocardial ischaemia.

A limitation of this study is the model of permanent coronary ligation, which allows no reperfusion. Based on the concern that ischaemia is an independent risk factor, this study addressed FOXC1 and TLRs in the context of ischaemia alone. Nevertheless, the ischaemia‐reperfusion model would be worth being investigated, as it is close to the clinic.

In summary, the present study shows that FOXC1, responsive as an ischaemia‐inducible TF, up‐regulates the expression of *Tlr* members in myocardial ischaemia. To the best of our knowledge, this is the first report on the regulation of *Tlr* expression by FOXC1.

## CONFLICT OF INTEREST

The authors confirm that there are no conflicts of interest.

## AUTHOR CONTRIBUTIONS

LL and QH designed the study. SZ, RY, JS, TG, RW and LP performed the experiments. SZ, RY, XP and XM analysed the data. SZ, LL and QH interpreted the results. SZ and LL wrote the manuscript. WY and QH reviewed and revised the manuscript.

## Data Availability

The data of this study are available from the corresponding author upon reasonable request.
