# Supplementary figures and images for: FOXC1 up‐regulates the expression of toll‐like receptors in myocardial ischaemia
Source: J Cell Mol Med. 2019 Sep 13;23(11):7566–80. doi: 10.1111/jcmm.14626 (PMC6815849; doi:10.1111/jcmm.14626)

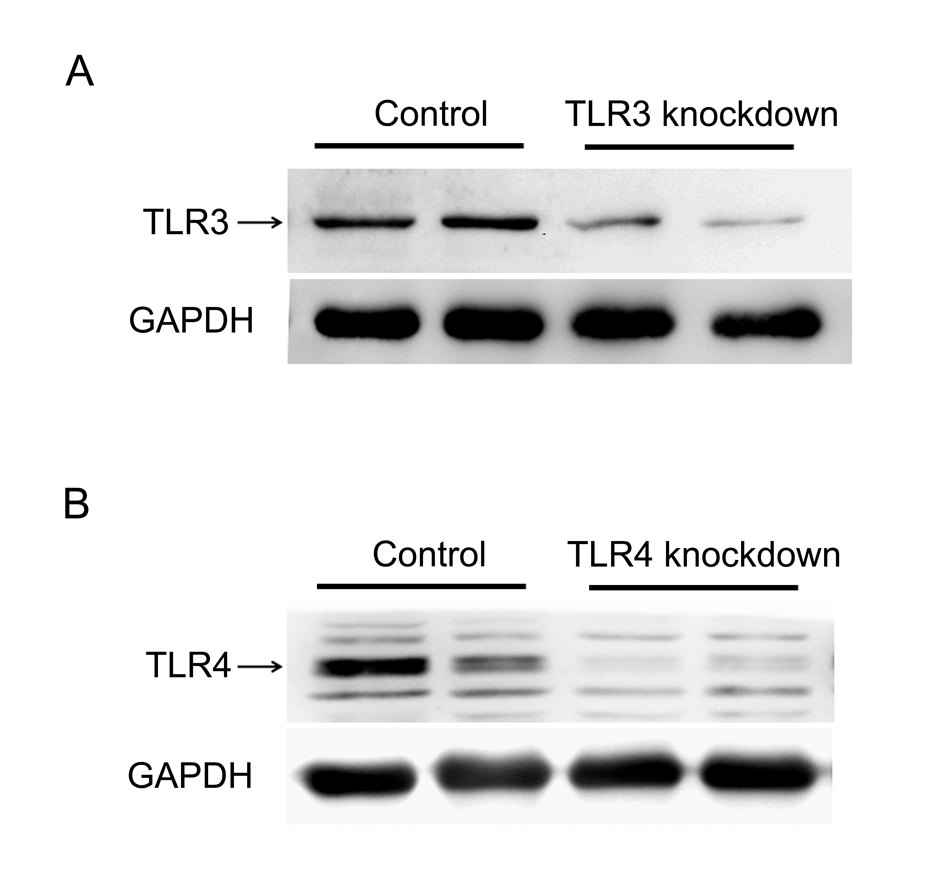

Supplement: Supplementary file 1 [file JCMM-23-7566-s001.tif]

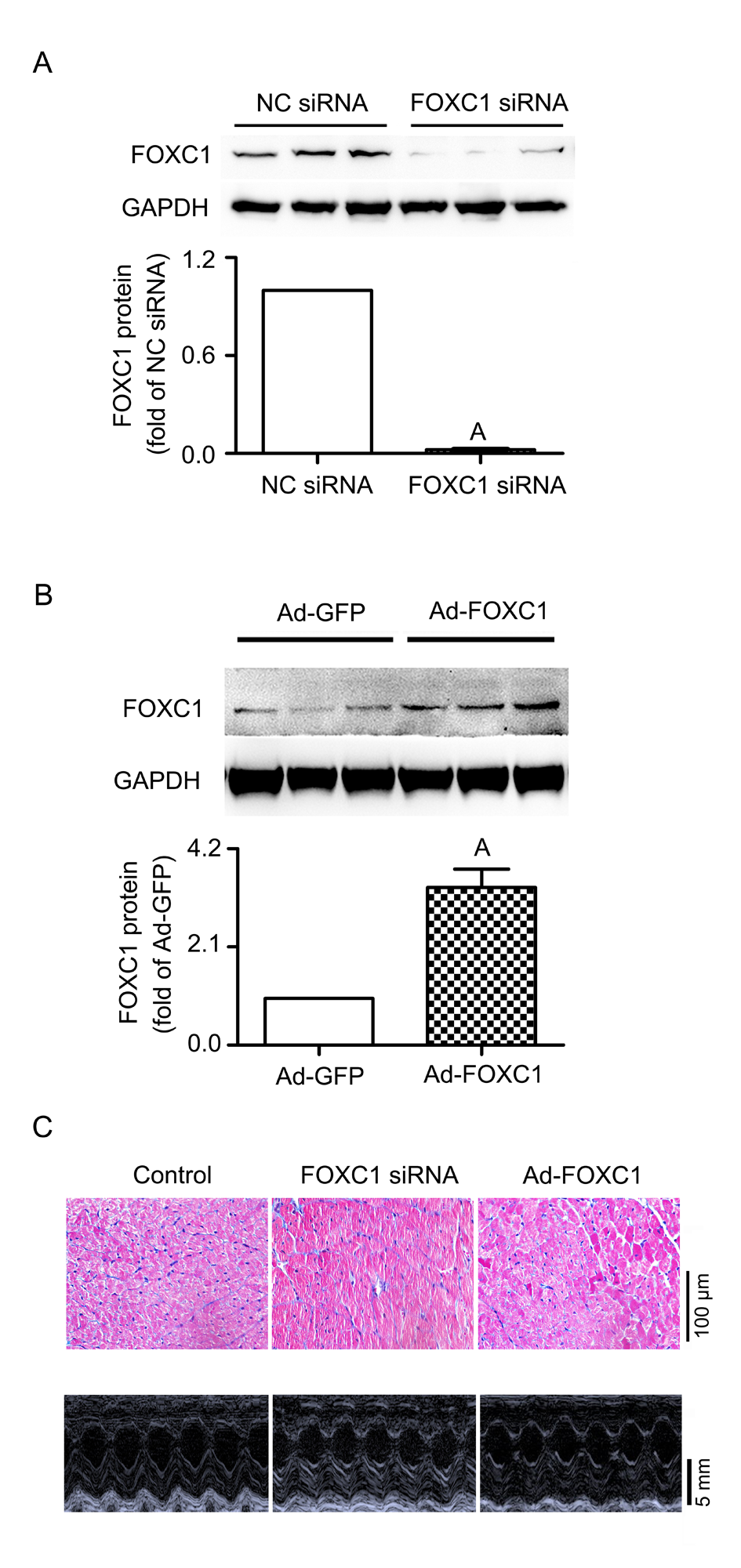

Supplement: Supplementary file 2 [file JCMM-23-7566-s002.tif]

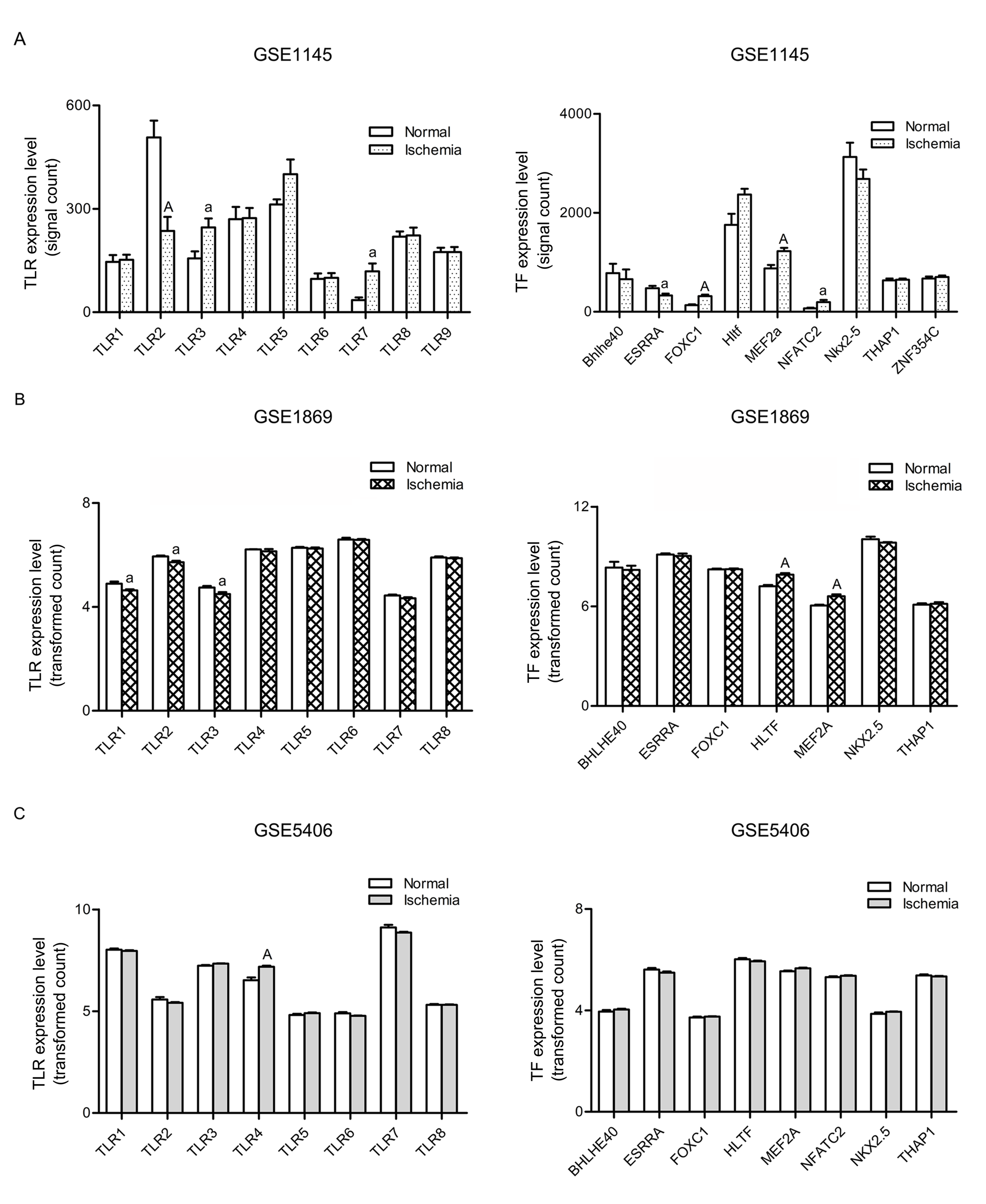

Supplement: Supplementary file 4 [file JCMM-23-7566-s004.tif]
