# Supplementary material for: FOXC1 up‐regulates the expression of toll‐like receptors in myocardial ischaemia
Source: J Cell Mol Med. 2019 Sep 13;23(11):7566–80. doi: 10.1111/jcmm.14626 (PMC6815849; doi:10.1111/jcmm.14626)
Supplement: Supplementary file 3 [file JCMM-23-7566-s003.docx]

Supplementary Table 1 Primers used for real-time PCR

| Gene | Forward primer (5' to 3') | Reverse primer (5’to 3’) |
| --- | --- | --- |
| TLR1 (mouse) | CAGGGCTGCTCTTATTTCTT | CGTTGTTTCCACATTGTTCA |
| TLR2 (mouse) | TATCCCCCAGTTCTATTTGT | GAT TTTCGCTGAGGTCTAAG |
| TLR3 (mouse) | CCTATGGATTCTTCTGGTGT | TCTTCTGAGTTGGTTGTGAG |
| TLR4 (mouse) | CAGTTTCAATCGCATAGAGA | CCAACACTAAGGAGGTATTC |
| TLR5 (mouse) | CGA AGACTGCGATGAAGAG | AGGGTGATGACGAGGAATAG |
| TLR6 (mouse) | GCTGTGAAGAATGGTAAAGT | GCAAACAAAGTGGAAACTC |
| TLR7 (mouse) | GAAGTTGGCTTTTGTCCTAA | GGCTCTTTGTTTTTGAACCT |
| TLR8 (mouse) | TCAGGAAATCGCATAGCATC | TCATCATCGTCTGTTGAGAG |
| TLR9 (mouse) | ATGGACGGGAACTGCTACTA | GACAGATGGGTGAGATTGCT |
| Bhlhe40 (mouse) | ACGTTGAAGCACGTGAAAGC | GAAGTACCTCACGGGCACAA |
| ESRRA (mouse) | AGTGTGAGATCACCAAGCGG | AAAGGCAAAGGGTCCACCTC |
| FOXC1 (mouse) | AAGGACGCAGTGAAGGACAA | CGTACCGTTCTCCGTCTTGAT |
| Hltf (mouse) | CAGGAAGGAGTGGTGTGGAC | CAAACAGGACCCCTCGTGAA |
| MEF2a (mouse) | AGTAGCGGAGACTCGGAATTG | ATGCATCGTACACAGCTCCT |
| NFATC2 (mouse) | GCCCCGCGACTCTATACGAA | GCCTTATGTGCGATCGGTTC |
| Nkx2-5 (mouse) | CTTCGTGAACTTTGGCGTCG | CGCCCTTCTCCTAAAGGTGG |
| THAP1 (mouse) | GGTGATCGTCCCCGTAACTG | TGTCCTTGTCGTAGCGGTTC |
| ZNF354C (mouse) | TGCTAGGAGAGAGCGTCTGG | TGACCAAATATGCGGACGCC |
| 18S (mouse) | CGTCTGCCCTATCAACTTTC | GGATGTGGTAGCCGTTTCT |
| TLR1 (rat) | TGTGGAGCCTTCAGTAGCCT | GCATCGGAATGGGTTGTT |
| TLR2 (rat) | CACATCCCCCAGTTCTATT | GATTTTCGCTGAGGTCTAAG |
| TLR3 (rat) | GAAAGGGCGTTCATCTTATC | GAGGGAAGGTCATCAGGTAT |
| TLR4 (rat) | GCCGTCTTCAATCTGACTAAT | ACACTGACCACCGATACACT |
| TLR5 (rat) | GTCTTGTTGCTGCTTGTTGAT | TTCTTCTTGTTGGCGGACT |
| TLR6 (rat) | GGTCCAACCTTATTGAATCT | TAAATGTCTCCCTGCTTATG |
| TLR7 (rat) | GTCCAAAGCCAATGTGTG | GCCCAGGTAGAGTGATTCAA |
| TLR8 (rat) | GCCTCCCAAACTACCAAG | TGTATTCTGATGGACGCAC |
| TLR9 (rat) | TCTCTCCACTCGCTGAACT | GCTGGTTTTGTTGATGCTC |
| Bhlhe40 (rat) | ACGTTGAAGCACGTGAAAGC | GAAGTACCTCACGGGCACAA |
| ESRRA (rat) | CTCCCAGTGGGTGATGCTTT | CTGGAGCCTGCTTGGAGTTA |
| FOXC1 (rat) | TTCAAGAAGAAGGACGCGGTG | GGACACGTACCGTTCTCCGT |
| Hltf (rat) | TGTGACACTGGAGAGAGGACA | CCAATCTGGTCAATCCAATTGCT |
| MEF2a (rat) | TGTAGCGGAGACTCGGAATTG | AAGGCTGCCGCTGAAATTGT |
| NFATC2 (rat) | ACGCCTTCTACTTTGGACCC | GTCCAGTCACCGTGGACATC |
| Nkx2-5 (rat) | CTTCGTGAACTTTGGCGTCG | CGCCCTTCTCCTAAAGGTGG |
| THAP1 (rat) | CCCACGGTGAGGTAATCGTC | ACAGGACTGCACCATCCTTC |
| ZNF354C (rat) | CGGAGATAGAAGCGTCGTCC | GCTCCTGTGCTGTCTCTAGC |
| 18S (rat) | GTTGAACCCCATTCGTGAT | GCTTATGACCCGCACTTACT |

Supplementary Table 2 Predicted FOXC1 binding sites in mouse TLR3 promoter

| Model ID | Model name | Score | Relative score | Start | End | Strand | predicted site sequence | site number in ChIP assay |
| --- | --- | --- | --- | --- | --- | --- | --- | --- |
| MA0032.1 | FOXC1 | 6.624 | 0.953 | -1295 | -1302 | 1 | aggaagta | 1 |
| MA0032.1 | FOXC1 | 6.624 | 0.953 | -590 | -597 | 1 | aggaagta | 2 |
| MA0032.1 | FOXC1 | 6.530 | 0.947 | -1439 | -1446 | 1 | agtatgta | 3 |
| MA0032.1 | FOXC1 | 6.530 | 0.947 | -1425 | -1432 | -1 | ggcatgta | / |
| MA0032.1 | FOXC1 | 6.501 | 0.946 | -655 | -662 | -1 | cctcagta | / |
| MA0032.1 | FOXC1 | 6.249 | 0.932 | -1282 | -1289 | -1 | gataagta | / |
| MA0032.1 | FOXC1 | 6.238 | 0.931 | -1443 | -1450 | 1 | ccccagta | / |
| MA0032.1 | FOXC1 | 6.179 | 0.928 | -1857 | -1864 | 1 | tcagagta | / |
| MA0032.1 | FOXC1 | 6.179 | 0.928 | -155 | -162 | 1 | cttgagta | / |
| MA0032.1 | FOXC1 | 6.045 | 0.920 | -1794 | -1801 | 1 | acaatgta | / |
| MA0032.1 | FOXC1 | 6.045 | 0.92 | -618 | -625 | 1 | agagtgta | / |
| MA0032.1 | FOXC1 | 5.823 | 0.908 | -946 | -953 | -1 | cctctgta | / |
| MA0032.1 | FOXC1 | 5.816 | 0.907 | -1141 | -1148 | 1 | ttaaagta | / |
| MA0032.1 | FOXC1 | 5.723 | 0.902 | -676 | -683 | 1 | tcaatgta | / |

Supplementary Table 3 Predicted FOXC1 binding sites in mouse TLR4 promoter

| Model ID | Model name | Score | Relative score | Start | End | Strand | predicted site sequence | site number in ChIP assay |
| --- | --- | --- | --- | --- | --- | --- | --- | --- |
| MA0032.1 | FOXC1 | 6.723 | 0.958 | -596 | -603 | 1 | agtcagta | 1 |
| MA0032.1 | FOXC1 | 6.624 | 0.953 | -1933 | -1940 | 1 | aggaagta | 2 |
| MA0032.1 | FOXC1 | 6.501 | 0.946 | -759 | -766 | 1 | cctcagta | 3 |
| MA0032.1 | FOXC1 | 6.501 | 0.946 | -137 | -144 | -1 | gcacagta | / |
| MA0032.1 | FOXC1 | 6.209 | 0.929 | -1217 | -1224 | 1 | tgtatgta | / |
| MA0032.1 | FOXC1 | 6.209 | 0.929 | -293 | -300 | -1 | gggatgta | / |
| MA0032.1 | FOXC1 | 5.986 | 0.917 | -360 | -367 | -1 | tgtgtgta | / |
| MA0032.1 | FOXC1 | 5.986 | 0.917 | -264 | -271 | -1 | tgtgtgta | / |
| MA0032.1 | FOXC1 | 5.823 | 0.908 | -1131 | -1138 | -1 | gccctgta | / |
| MA0032.1 | FOXC1 | 5.816 | 0.907 | -1052 | -1059 | -1 | ctgaagta | / |
| MA0032.1 | FOXC1 | 5.816 | 0.907 | -887 | -894 | 1 | ttaaagta | / |
| MA0032.1 | FOXC1 | 5.782 | 0.905 | -1431 | -1438 | 1 | agactgta | / |
| MA0032.1 | FOXC1 | 5.723 | 0.902 | -1425 | -1432 | -1 | attatgta | / |
| MA0032.1 | FOXC1 | 5.723 | 0.902 | -994 | -1001 | 1 | attatgta | / |

Supplementary Table 4 Primers used for ChIP in mouse TLR3 and TLR4 promoters

| FOXC1 binding site | Forward primer (5' to 3') | Reverse primer (5’to 3’) |
| --- | --- | --- |
| site 1 in TLR3 promoter | TAGGTAAATGTCAGGTGGAT | TTCATTGAGGAAGGGTCT |
| site 2 in TLR3 promoter | AGGCTTCTGATTGCATAG | GCCTGATAAGTGCCTCTG |
| site 3 in TLR3 promoter | ACCATACAGCAGCCGATTC | TTGGCAAGATTTCACCTC |
| site 1 in TLR4 promoter | GATGAGCTATCTTCCCAACT | GGGTCCCTTCATTGTTCT |
| site 2 in TLR4 promoter | ATAGCCAAGGAATCAGTA | CAGGAAAGATAGGAGCAG |
| site 3 in TLR4 promoter | TGCTCTGGGAGACTTGGG | TGGTGAAGGCAGGGAACT |

Supplementary Table 5 Parameters of echocardiography

|  | n | LVEDD  (mm) | LVESD  (mm) | Fractional shortening (%) |
| --- | --- | --- | --- | --- |
| NC siRNA + sham | 8 | 3.18±0.05 | 1.51±0.04 | 52.61±1.35 |
| FOXC1 siRNA + sham | 8 | 3.17±0.10 | 1.51±0.02 | 52.27±1.02 |
| NC siRNA + MI | 10 | 3.68±0.03 ** | 2.84±0.10 ** | 22.55±1.23 ** |
| FOXC1 siRNA + MI | 10 | 3.64±0.03 ** | 2.64±0.03 **^##^ | 27.63±0.71 **^#^ |
| Ad-GFP + sham | 7 | 3.16±0.03 | 1.52±0.01 | 51.94±0.56 |
| Ad-FOXC1 + sham | 7 | 3.18±0.05 | 1.52±0.01 | 52.13±0.83 |
| Ad-GFP + MI | 8 | 3.99±0.15 ** | 3.07±0.12 ** | 22.88±0.74 ** |
| Ad-FOXC1 + MI | 8 | 4.08±0.12 ** | 3.54±0.10 **^#^ | 13.24±0.58 **^##^ |

*P<0.05, **P<0.01 vs. respective sham; ^#^P<0.05, ^##^P<0.01 vs. respective MI.
