# Supplementary material for: FOXC1 up‐regulates the expression of toll‐like receptors in myocardial ischaemia
Source: J Cell Mol Med. 2019 Sep 13;23(11):7566–80. doi: 10.1111/jcmm.14626 (PMC6815849; doi:10.1111/jcmm.14626)
Supplement: Supplementary file 5 [file JCMM-23-7566-s005.docx]

**Supplementary figure legends**

**Supplementary Figure 1. Expression of toll-like receptors (TLRs) and the 9 selected transcription factors (TFs) in human heart.** Three publicly available datasets containing the expression data of TLRs and selected TFs in the left ventricular tissue of patients with ischemic cardiomyopathy were found on Gene Expression Omnibus (GEO), under the GEO accession numbers GSE1145 (Martina et al.), GSE1869 (Kittleson et al.) and GSE5406 (Cappola et al.). The expression levels, expressed as count data for GSE1145 and transformed count data form Robust Multi-Array Analysis (RMA) for GSE1869 and GSE5406, were compared between the normal and ischemia groups. ^a^*P* < 0.05, ^A^*P* < 0.01 vs. respective normal group.

**Supplementary Figure 2. Validation of the antibodies for TLR3 and TLR4.** The siRNAs against TLR3/4 were used to knockdown their expression in H9c2 cells, and then western blotting was performed to identify the protein bands for TLR3/4, as indicated by arrows.

**Supplementary Figure 3. Histology and heart function of sham-operated mouse heart subjected to FOXC1 interventions.** (A) The siRNA against FOXC1 significantly reduced FOXC1 proteins in left ventricular tissue. (B) The adenovirus vector expressing FOXC1 (Ad-FOXC1) significantly increased FOXC1 proteins in left ventricular tissue. (C) The Masson’s trichrome staining (upper panel) and echocardiography (lower panel) showed no changes in histology and heart function for sham-operated (normal) heart receiving either FOXC1 siRNA or Ad-FOXC1.
